# Toll-like Receptor 9 Can be Activated by Endogenous Mitochondrial DNA to Induce Podocyte Apoptosis

**DOI:** 10.1038/srep22579

**Published:** 2016-03-03

**Authors:** Wenduona Bao, Hong Xia, Yaojun Liang, Yuting Ye, Yuqiu Lu, Xiaodong Xu, Aiping Duan, Jing He, Zhaohong Chen, Yan Wu, Xia Wang, Chunxia Zheng, Zhihong Liu, Shaolin Shi

**Affiliations:** 1 National Clinical Research Center of Kidney Diseases, Jinling Hospital, Nanjing University School of Medicine, Nanjing, China

## Abstract

Toll-like receptor 9 (TLR9) senses bacterial DNA characteristic of unmethylated CpG motifs to induce innate immune response. TLR9 is *de novo* expressed in podocytes of some patients with glomerular diseases, but its role in podocyte injury remains undetermined. Since TLR9 activates p38 MAPK and NFkB that are known to mediate podocyte apoptosis, we hypothesized that TLR9 induces podocyte apoptosis in glomerular diseases. We treated immortalized podocytes with puromycin aminonucleosides (PAN) and observed podocyte apoptosis, accompanied by TLR9 upregulation. Prevention of TLR9 upregulation by siRNA significantly attenuated NFκB p65 or p38 activity and apoptosis, demonstrating that TLR9 mediates podocyte apoptosis. We next showed that endogenous mitochondrial DNA (mtDNA), whose CpG motifs are also unmethylated, is the ligand for TLR9, because PAN induced mtDNA accumulation in endolysosomes where TLR9 is localized, overexpression of endolysosomal DNase 2 attenuated PAN-induced p38 or p65 activity and podocyte apoptosis, and DNase 2 silencing was sufficient to activate p38 or p65 and induce apoptosis. In PAN-treated rats, TLR9 was upregulated in the podocytes, accompanied by increase of apoptosis markers. Thus, *de novo* expressed TLR9 may utilize endogenous mtDNA as the ligand to facilitate podocyte apoptosis, a novel mechanism underlying podocyte injury in glomerular diseases.

Toll-like receptor (TLR) family consists of the members, TLR1 though TLR12, which function to recognize pathogen associated molecular patterns (PAMPs) and trigger immune response in the body. They are mainly expressed in immune cells. Upon PAMP ligand binding, TLRs trigger a cascade of molecular events, leading to activation of NFκB signaling and expression of downstream targets, eg., IL-12. TLRs can induce p38 MAPK signaling as well^1^. Among these TLRs, TLR9 is special in that it is localized in endolysosomes^2^ and has its unique ligand, the unmethylated CpG motifs of bacterial DNA. Bacterial DNA is trafficked through endocytosis to endolysosomes where it acts on TLR9 to elicit NFκB and p38 MAPK signaling in immune cells. Recently, mitochondrial DNA (mtDNA), which is also unmethylated at CpG motifs, has been shown to be taken up by immune cells in which it induces inflammatory response through activating TLR9^3^.

Podocytes are renal intrinsic cells, functioning as part of glomerular filtration barrier. Their dysfunction or injury changes glomerular permeability, leading to proteinuria, and their loss can result in glomerular damage and glomerulosclerosis^4^,^5^,^6^. Podocyte apoptosis is one of the major ways by which podocytes are lost^7^,^8^. At present, the mechanism underlying podocyte apoptosis is incompletely understood. Studies have implicated NFκB signaling in podocyte injury and proteinuria^9^,^10^. Moreover, p38 MAPK signaling has also been demonstrated to mediate podocyte injury and apoptosis^8^,^11^. In the research area of podocytes, puromycin aminonucleosides (PAN) is a commonly-used model of podocyte injury. It is known that PAN can induce nephrotic syndrome in rats and directly injure podocytes independently of immune process^6^,^12^,^13^,^14^. It is also known that PAN activates NFκB and p38 MAPK to induce apoptosis in cultured podocytes^11^,^15^. Therefore, PAN-induced podocyte injury is a good model for the study of molecular mechanisms underlying podocyte apoptosis.

Recently, TLR9 has been shown to be *de novo* expressed in podocytes in the patients with lupus nephropathy and several other glomerular diseases^16^,^17^,^18^,^19^. Since TLR9 can trigger p38 and NFκB signaling which are known to be involved in podocyte apoptosis, we speculated that *de novo* expressed TLR9 in the podocytes of patients with these diseases may facilitate podocyte apoptosis. We further hypothesized that the ligand of TLR9 in the podocytes might be the endogenous mitochondrial DNA. We tested these hypotheses by *in vitro* model of PAN-induced podocyte injury and in an *in vivo* PAN treated rat model.

## Results

### PAN upregulates TLR9 expression and activates NFκB and p38 MAPK in human immortalized podocytes

**Figure 1:**
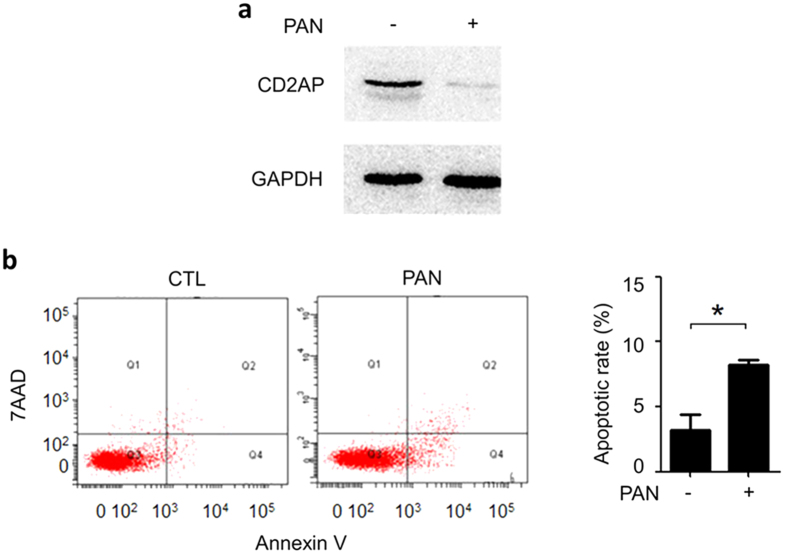
PAN induced podocyte injury. (**a**) Immunoblotting of CD2AP in cultured human podocytes treated with 50 μg/ml of PAN for 24 h revealed CD2AP downregulation. (**b**) Annexin V flow cytometry analysis of the same podocytes, showing that PAN induced significant podocyte apoptosis. *P < 0.01.

PAN is commonly used to induce podocyte injury. We treated human immortalized podocytes in culture with PAN (50 μg/ml for 24 h, a condition that is most frequently used in literatures), and observed significant CD2AP downregulation (Fig. 1a) and apoptosis of the podocytes (8.40% ± 0.23%; p < 0.05 versus control) (Fig. 1b), indicating that PAN-induced podocyte injury model was successful.

**Figure 2:**
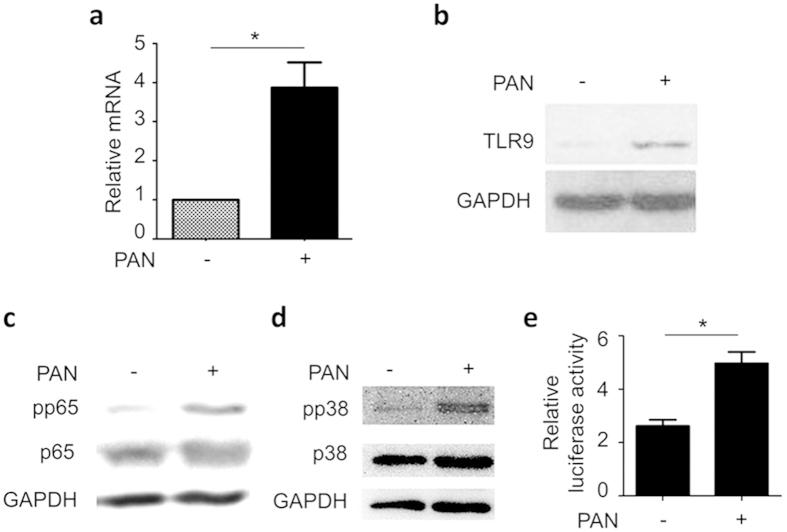
PAN upregulated TLR9 expression and activated NFκB and p38 MAPK in human podocytes. (**a**) qPCR analysis of TLR9 mRNA in the podocytes treated with PAN for 12 h, showing that TLR9 mRNA was significantly upregulated *P < 0.01. (**b**) Immunoblotting of TLR9 of the same cells also revealed upregulation of TLR9 protein. (**c**,**d**) PAN enhanced p65 (**c**) and p38 (**d**) phosphorylation. (**e**) Luciferase reporter assay by using NFκB responsive reporter luciferase construct further confirmed that PAN activated NFκB signaling in the podocytes. *P < 0.05.

We examined TLR9 mRNA in the podocytes by qPCR and found that PAN treatment significantly upregulated TLR9 mRNA (Fig. 2a). Consistently, immunoblotting showed an increase of TLR9 protein in the cells (Fig. 2b). Thus, it appeared that PAN-induced podocyte injury is an appropriate model to investigate the role of TLR9 in podocyte injury.

We next examined the signaling pathways downstream of TLR9, including NFκB and p38 MAPK, and found that the phosphorylations of both NFκB p65 and p38 were significantly increased by PAN (Fig. 2c,d). To further confirm that NFκB signaling was enhanced, we performed luciferase reporter assay using a luciferase expressing construct with NFκB-responsive promoter, and found increased luciferase activity in the cells after PAN treatment (Fig. 2e). Consistently, downstream components of NFκB signaling, IL12, IFN-A2, and CYCS were also increased (Supplementary Fig. S1).

### TLR9 is functional in human podocytes

**Figure 3:**
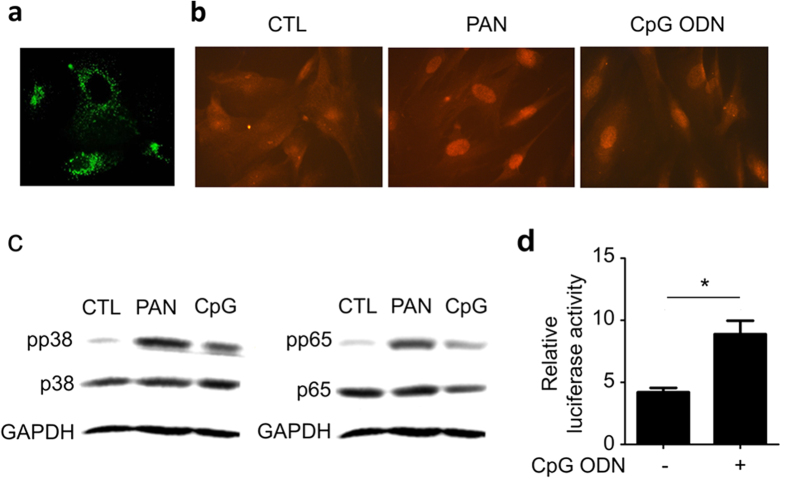
TLR9 in human podocytes is functional. (**a**) Green fluorescence-labelled ODN was taken up by the cells and delivered to endolysosomes. (**b**) Immunofluorescent staining of phosphorylated p65 (pp65) showing its nuclear accumulation in the cells treated as indicated. (**c)** Immunoblotting of pp38 (left) and pp65 (right) in the cells treated as indicated, showing that PAN and CpG ODN activated NFκB and p38 signaling pathways. (**d**) Luciferase reporter assay demonstrating that CpG ODN is capable of acting on TLR9, leading to NFκB activation in podocytes. *P<0.05.

To determine whether TLR9 is functional in human podocytes and capable of inducing NFκB or p38 signaling upon ligand binding, we stimulated the cells with CpG ODN. Firstly, we added fluorescently-labelled CpG ODN to podocytes in culture and observed a quick uptake of the ODN by the cells and delivery to endolysosomes (Fig. 3a). The endolysosomal identity of the stained organelles was confirmed by co-staining with endolysosomal marker, lysotracker (Supplementary Fig. S2). Next, we treated podocytes with PAN or CpG ODN followed by immunofluorescence staining of phosphorylated NFκB p65 (pp65), and observed a significant nucleic accumulation of pp65 in the cells in either treatment (Fig. 3b). Immunoblotting showed that both PAN and CpG ODN can activate p38 and p65 (Fig. 3c). Consistently, CpG ODN treatment caused increased apoptosis in the cells (Supplementary Fig. S3). Finally, we performed luciferase reporter assay using NFκB-responsive luciferase expression construct and found, as expected, that CpG ODN increased luciferase expression in the cells (Fig. 3d).

To further support that TLR9 is functional in podocytes, we examined the mRNA expression of the components in TLR9 pathway from microarray gene expression profile of human podocytes, and found significant intensity readings (Supplementary Fig. S4a). Immunoblotting of several of them, including IRAK1 and TRAF6, confirmed the results from microarray (Supplementary Fig. 4b).

### Prevention of TLR9 upregulation eliminates PAN-induced NFκB and p38 activation and podocyte apoptosis

**Figure 4:**
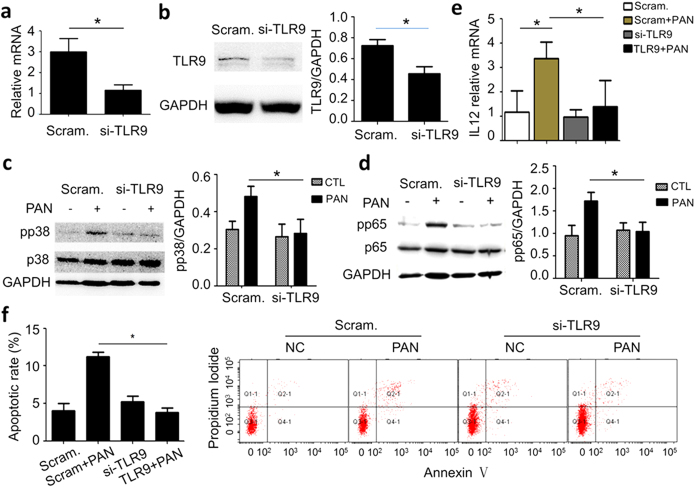
Prevention of TLR9 upregulation by siRNA eliminated PAN-induced p38 and NFκB signaling and podocyte apoptosis. (**a**) qRT-PCR analysis showing that TLR9 siRNA transfection resulted in TLR9 mRNA reduction in podocytes. (**b**) Immunoblotting of TLR9 in PAN-treated podocytes with either scramble control or si-TLR9 treatment, showing that TLR9 knockdown prevented PAN-induced upregulation of TLR9. (**c**,**d**) Immunoblotting showing that TLR9 knockdown prevented phosphorylation of p38 (**c**) and p65 (**d**) in PAN treatment. (**e**) qRT-PCR analysis of IL-12 in the cells indicated shows that si-TLR9 mitigated IL-12 upregulation induced by PAN. (**f**) Annexin V flow cytometry demonstrating that TLR9 knockdown prevented PAN-induced podocyte apoptosis. *P < 0.05. Please note that the bar graphs on the right in Panel (**b**–**d**) show the quantification of the results from at least three independent experiments. *P < 0.05.

To determine the role of TLR9 in PAN-induced podocyte injury, we knocked down TLR9 by siRNA in the podocytes prior to PAN treatment. qRT-PCR analysis of TLR9 in the podocytes treated with siRNA against TLR9 (si-TLR9) showed that TLR9 mRNA level was significantly reduced compared with scramble control (Fig. 4a). As expected, si-TLR9 significantly mitigated PAN-induced TLR9 protein upregulation (Fig. 4b).

Next, we examined phosphorylation (activation) of p38 and NFκB p65 in the cells. We found that si-TLR9 treatment almost completely abolished PAN-induced p38 phosphorylation (Fig. 4c), as well as p65 phosphorylation in the treatment of PAN (Fig. 4d). Consistently, the upregulation of downstream IL-12 was also prevented by si-TLR9 (Fig. 4e). These results demonstrated that TLR9 mediates PAN-induced p38 or p65 signaling activation.

Since PAN is known to induce podocyte apoptosis, a common pathway of various types of podocyte injury, we investigated the role of TLR9 in podocyte apoptosis induced by PAN. We found that PAN-induced podocyte apoptosis was completely abolished by si-TLR9 (Fig. 4f) as shown by flow cytometry analysis of the cells.

### Overexpression of TLR9 elevated basal levels of p38 and p65 phosphorylation and apoptosis in podocytes

As an opposite approach, we overexpressed TLR9 in podocytes and examined its effect on p38 and p65 phosphorylation and podocyte apoptosis. We found that TLR9 overexpression increased the basal levels of both pp38 and pp65 in the absence of PAN (Supplementary Fig. S5a,b). However, it did not further enhance the levels of pp38 and pp65 in PAN treatment (Supplementary Fig. S5a,b). Finally, TLR9 overexpression neither significantly induced podocyte apoptosis in the absence of PAN nor aggravated the apoptosis in the presence of PAN (data not shown). These observations suggest, at least under the present experimental condition, that p38 or p65 phosphorylation/activation is required but not sufficient for podocyte apoptosis; and that TLR9 overexpression might be capable of activating p38 or p65 but not the other components that are also required for podocyte apoptosis in contrast to PAN which can activate all necessary factors for apoptosis.

### PAN induces mtDNA accumulation in the endolysosomes

Having clearly demonstrated a role of TLR9 in podocyte injury, we next asked what the ligand was for TLR9 activation in the experimental system. Mitochondria are considered to originate from bacteria in evolution and their mtDNA retains many features of bacterial DNA, including unmethylated CpG motifs. We therefore speculated that endogenous mtDNA of the podocytes might be the ligand for TLR9. It is possible that PAN may damage mitochondria and the damaged mitochondria are delivered by autophagy to endolysosomes where the introduced mtDNA could act on TLR9.

**Figure 5:**
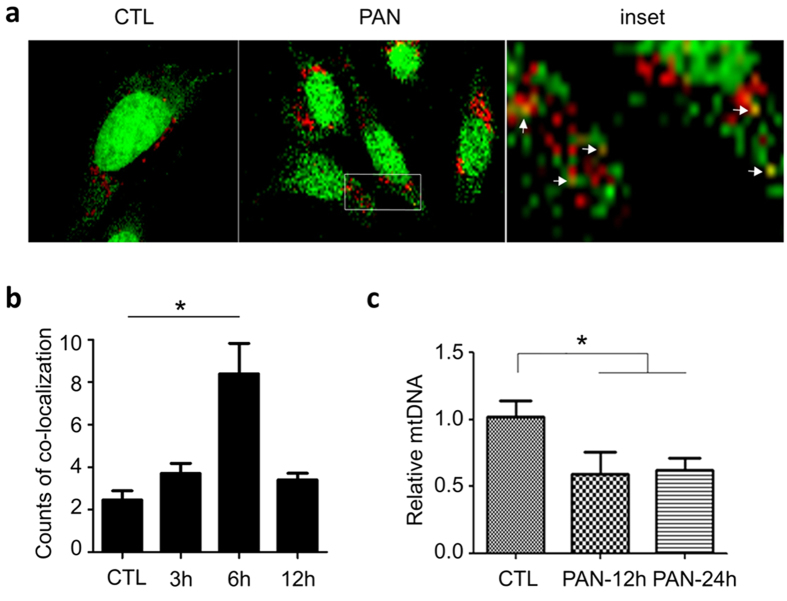
PAN-induced mtDNA accumulation in endolysosomes. (**a**) Double staining with Picogreen and Lysotracker (red) showing that PAN-induced mtDNA trafficking to endolysosomes. (**b**) Quantification of the co-localization of Picogreen and Lysotracker fluorescence. *P < 0.05 versus 0 h. (**c**) qPCR analysis showing that the overall intracellular mtDNA was decreased by PAN, suggesting that PAN treatment induces mtDNA translocation to endolysosomes for degradation and TLR9 activation. *P < 0.05.

To prove this, we treated podocytes with PAN, followed by co-staining with Picogreen (for mtDNA) and Lysotracker (for endolysosomes). We observed a significant increase of co-localization of the green and red fluorescences (Fig. 5a,b), indicating that PAN promoted mtDNA translocation to endolysosomes. Consistently, we found that the overall intracellular mtDNA content in the cells decreased in the treatment of PAN as shown by qPCR analysis (Fig. 5c), supporting that mtDNA is delivered to endolysosomes where it may activate TLR9 while being degraded in the treatment of PAN.

### DNase 2 overexpression mitigated p38 and p65 activation and apoptosis in the podocytes treated with PAN

**Figure 6:**
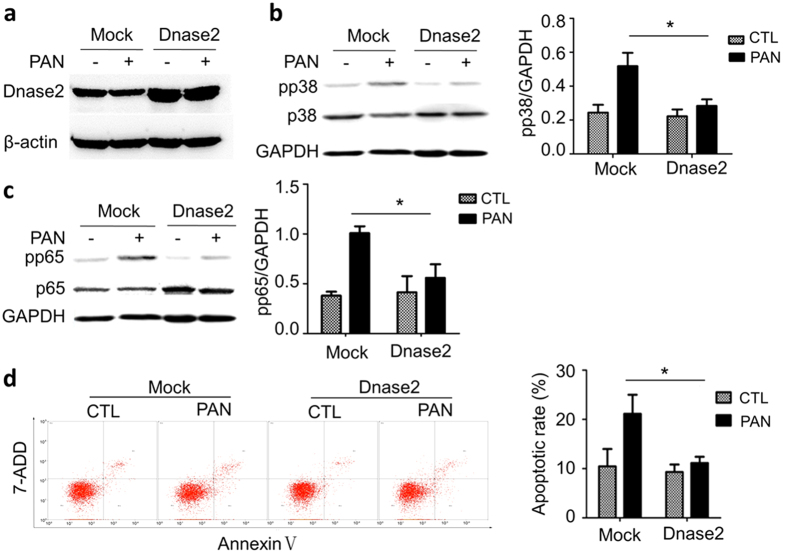
The effects of DNase 2 overexpression on podocytes. (**a**) Immunoblotting shows that DNase 2 protein was increased after transfection of DNase 2 expression plasmid. (**b**) Immunoblotting of pp38 in the cells overexpressing DNase 2 in the absence or presence of PAN. (**c**) Immunoblotting of pp65 in the same cells in B. (**d**) Annexin V flow cytometry analysis showing that DNase 2 overexpression prevented podocyte apoptosis induced by PAN. The bar graphs on the right in Panel (**b**,**c**) and d show the quantification of the results from at least three independent experiments. *P < 0.05.

If mtDNA is the ligand for TLR9 in podocytes, we would expect that overexpression of DNase 2, the lysosomal specific DNase, in the cells would eliminate the accumulation of mtDNA in endolysosomes, thereby preventing p38 or p65 activation. We transfected DNase 2 expressing construct in podocytes, and observed increased protein of DNase 2 in the cells (Fig. 6a). Immunoblotting showed that PAN significantly increased pp38 in the cells; however, this increase was prevented by DNase 2 overexpression (Fig. 6b). DNase 2 overexpression also prevented PAN-induced pp65 upregulation (Fig. 6c). Consistently, Flow cytometry analysis of the cells following Annexin V staining demonstrated that DNase 2 overexpression prevented PAN-induced apoptosis (Fig. 6d).

### DNase 2 knockdown enhances podocyte p38 and p65 activities and apoptosis in the treatment of PAN

**Figure 7:**
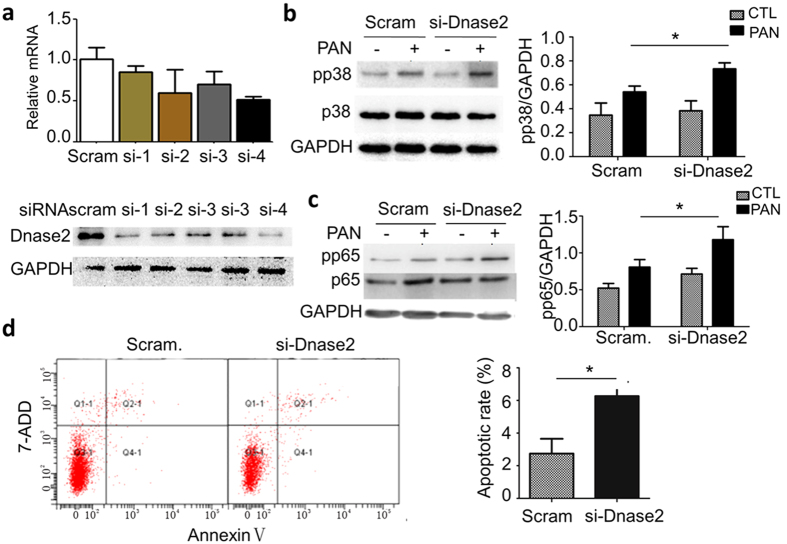
The effects of DNase 2 knockdown in podocytes. (**a**) qPCR analysis of the DNase 2 mRNA (top) and protein (bottom) levels after transfection of DNase 2 siRNAs. This experiment identified #4 siRNA (si-4) as the most efficient one, which was used in the study. (**b**) Immunoblotting of pp38 in the cells treated with control or the si-4 construct in the absence or presence of PAN. *P < 0.05. (**c**) Immunoblotting of pp65 in the cells similarly treated. *P < 0.05. (**d**) Flow cytometry analysis demonstrated that DNase 2 knockdown was sufficient to increase basal level apoptosis of podocytes (without PAN treatment). *P < 0.05. The bar graphs on the right in Panel b and c show the quantification of the blots from three independent experiments. *P < 0.05.

We expected opposite effects of DNase 2 knockdown compared to overexpression on podocytes. We first screened for effective siRNAs and found one (si-4) that can significantly reduce both DNase 2 mRNA and protein (Fig. 7a). When this siRNA was transfected into podocytes followed by PAN treatment, we observed that PAN-induced p38 phosphorylation was further increased (Fig. 7b). DNase 2 knockdown also resulted in additional increase of p65 phosphorylation in the treatment of PAN (Fig. 7c). Lastly, DNase 2 knockdown was found to be sufficient to induce podocyte apoptosis (Fig. 7d).

### TLR9 is upregulated in the podocytes of PAN-rats

**Figure 8:**
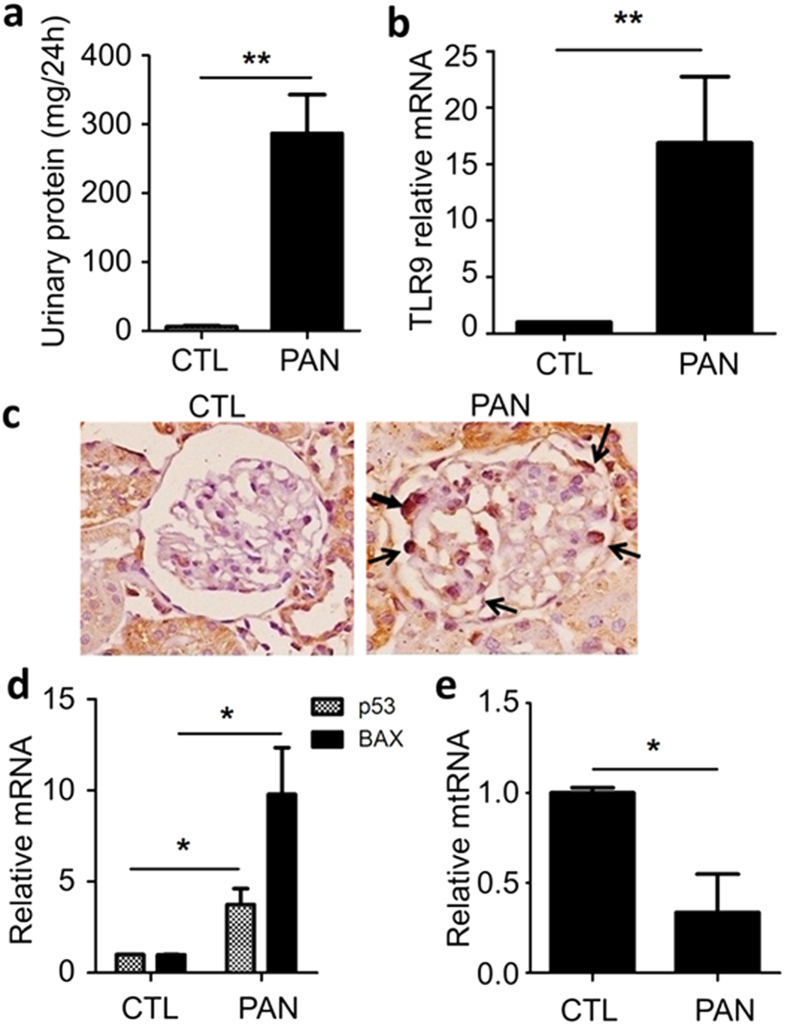
Characterization of PAN-treated rats. (**a**) Marked proteinuria developed at day 6 following PAN injection. n = 6 in both control and PAN group. (**b**) qRT-PCR analysis of TLR9 mRNA in the glomeruli of control rats and PAN-treated rats. n = 6 in each group. (**c**) Immunohistochemical staining of TLR9 in the glomeruli of the rats, showing TLR9 proteins in the podocytes (arrows). n = 6 in each group. (**d**) qRT-PCR analysis of p53 and Bax in the glomeruli of the rats. (**e**) qPCR analysis of mtDNA in glomeruli of the rats. n = 6 in each group. *P < 0.05, **P  < 0.01.

To investigate whether TLR9 plays a role in podocyte apoptosis *in vivo*, we examined TLR9 expression in the podocytes of the rats treated with PAN. At day 6 following PAN treatment when marked proteinuria had developed (Fig. 8a), the rats were sacrificed and the glomeruli were isolated for TLR9 mRNA qPCR analysis. We found that TLR9 mRNA was significantly increased compared with control rats (Fig. 8b). TLR9 upregulation in podocytes of the PAN-treated rats was further confirmed by IHC staining of TLR9 on the renal tissue of the rats (Fig. 8c). qPCR analysis of the glomeruli showed that two podocyte apoptosis markers, p53 and Bax, were both significantly upregulated (Fig. 8d). We also quantified mtDNA content in the glomeruli of PAN-treated rats and found it was significantly decreased (Fig. 8e), suggesting that mtDNA might have been delivered along with damaged mitochondria to lysosomes where it could activate TLR9.

## Discussion

Podocytes are part of glomerular filtration barriers^20^, and their dysfunction, injury or loss is thought to initiate glomerular damage and disease^5^. Understanding the process and identifying the molecular drivers of podocyte injury are important with regard to prevention and treatment of glomerular diseases. Since studies have shown that TLR9 is *de novo* expressed in the podocytes or glomeruli in Lupus nephropathy and several other glomerular diseases, including IgAN and FSGS^17^,^18^,^19^,^21^ and that TLR9 activation can induce NFκB or p38 MAPK signaling, two signaling pathways that are known to be involved in podocyte apoptosis^8^,^10^,^11^,^22^, we hypothesized that the *de novo* expressed TLR9 may promote podocyte apoptosis in the glomerular diseases.

To investigate this issue, an appropriate experimental model is required. PAN-induced podocyte injury is one of the most commonly used models for podocyte injury. PAN can induce podocyte apoptosis and cytoskeletal damage, resembling the podocyte manifestations of the patients with glomerular diseases. PAN has been shown to activate p38 MAPK and NFκB and induce podocyte apoptosis^15^,^23^. Importantly, we found that PAN can markedly upregulate TLR9, making it an ideal model to determine the role of TLR9 in podocye injury.

We used siRNA to prevent TLR9 upregulation in the treatment of PAN and examined its effects on p38, p65 and apoptosis in the cells. We found that both p38/p65 activation and podocyte apoptosis were mitigated by TLR9 knockdown, demonstrating that TLR9 mediates PAN-induced p38/p65 activation and apoptosis in podocytes. We also performed TLR9 overexpression in cultured podocytes, expecting that the p38/p65 activation and podocyte apoptosis would be aggravated in the presence or absence of PAN. However, we found that TLR9 overexpression only increased p38/p65 phosphorylation in the absence of PAN (basal level) but did not further increase it significantly in the treatment of PAN. We did not observe significant effect of TLR9 overexpression on apoptosis in the treatment with or without PAN. Overall, TLR9 overexpression had little effect on podocyte injury. We speculate that one reason underlying this observation could be the limited supply of ligand, in another word, the endogenous mtDNA introduced into endolysosomes might have already been saturated by the pre-existing and/or PAN-induced TLR9, making the overexpressed exogenous TLR9 useless.

In seeking the ligand for TLR9 in our experimental system, we hypothesized that endogenous mtDNA was most likely the ligand because mitochondrial DNA is also unmethylated at the CpG motifs and recent studies have shown that it can serve as TLR9 ligand^24^. Because our cell culture system was free of any bacteria or viruses, hence their DNA. By using Picogreen, we traced the mtDNA and observed that PAN treatment caused mtDNA accumulation in endolysosomes. Overexpression of DNase 2 eliminated PAN-induced p65/p38 activation and podocyte apoptosis. On contrary, DNase 2 knockdown by siRNA had opposite effects. Together, these data suggest that endogenous mtDNA can act as a ligand for the *de novo* expressed TLR9 to exert pathogenic effects in podocytes. Our findings have once again demonstrated that mtDNA is TLR9 ligand and pathogenic in diseases. Recently, endogenous mtDNA has been shown to be capable of inducing cardiomyocyte injury^25^. This study, together with our present work, strongly suggest that endogenous mtDNA-TLR9 pathway may be a common mechanism underlying the injury of many cell types in the organisms.

It would be interesting to understand how TLR9 is upregulated in podocytes of only part of the patients with lupus nephropathy or several other glomerular diseases. Several studies have shown that TLR9 can be induced in endothelial cells through ROS^26^,^27^ and one of the prominent effects of PAN on podocytes is known to be oxidation stress^28^,^29^,^30^,^31^, these suggest that TLR9 upregulation in PAN-treated podocytes could be due to ROS stress in the cells. This can be tested by using anti-oxidative reagents and examining whether addition of anti-oxidant would eliminate TLR9 upregulation by PAN. The mechanism of TLR9 *de novo* expression in podocytes in the patients remains unknown and warrant further investigation for therapeutic target identification.

Inhibition of TLR9 activity may help halt the progression of glomerular diseases in the patients that have *de novo* TLR9 expression in the podocytes. The identification of endogenous mtDNA as TLR9 ligand may have provided an alternative therapeutic approach that is based on disrupting the interaction between mtDNA and TLR9. To block this pathway, prompt elimination of mtDNA in endolysosomes by, eg., enhanced DNase 2 activity, might be effective. Inhibitory CpG ODN that binds to but does not stimulate TLR9 may be another approach to block the signaling pathway.

TLR family consists of more than 10 members, each of which has distinct ligands. We wondered whether the receptors other than TLR9 are also regulated in expression by PAN. qRT-PCR analysis showed that TLR4 and TLR7 mRNAs were also upregulated by PAN in podocytes (Supplemental Fig. S6). However, we do not think the upregulation of TLR4 or TLR7 contributed to the results that we obtained throughout the study. This is because TLR7 upregulation occurred essentially at 48 h after PAN treatment, prior to which the PAN and TLR9 effects had occurred. In contrast, TLR4 upregulation by PAN took place much earlier, but TLR4 uses distinct ligands for activation, eg., Liposaccharides (LPS), which does not exist in our experimental system.

TLR9 upregulation in podocytes or glomeruli has been reported in lupus nephropathy^16^,^17^,^19^. In addition to LN, TLR9 upregulation was also reported in some cases of other glomerular diseases, including FSGS, MCD, membranous nephropathy, as well as haemolytic uraemic syndrome (HUS) and polyoma virus nephropathy^16^,^18^. We have also examined TLR9 expression in the glomeruli of FSGS patients and found 2 cases (out of 6) in which TLR9 was upregulated in podocytes (Supplemental Fig. S7). It appears that TLR9 is turned on in podocytes of a quite broad spectrum of glomerular disease, although this takes place in only a portion of the patients in each disease.

In conclusion, our present study has implicated TLR9 and endogenous mtDNA in podocyte injury and suggests TLR9 and mtDNA as therapeutic targets for the patients with TLR9 *de novo* expression in podocytes. Further studies, especially those using animals to confirm the *in vivo* role of TLR9 and mtDNA in podocyte injury, is warranted.

## Materials and Methods

### Materials

#### Reagents

The conditionally immortalized human podocytes (HPC) was a gift from Dr. Moin Saleem at Bristol University, UK. The cells were cultured in RPMI 1640 containing 10% fetal bovine serum and pennicilin/streptomycin (100 U/ml of each) (Gibco, USA) and 1% insulin-transferrin-selenium (ITS, Invitrogen). Other reagents used in the studies were as follows: podocyte transfection reagent (LipofecTAMINE 2000, Invitrogen); plasmid DNA extraction kit (Purelink HiPure Plasmid DNA Purification Kit, Invitrogen), antibodies against total p65, phospho-p65, total p38 and phospho-p38 (Cell Signaling Technologies), TLR9 and CD2AP (Abcam), podocin (Sigma), GAPDH (Kangchen), anti-rabbit IgG (Bioworld); puromycin aminonucleosides (Sigma); Alexa Fluor® 647 Annexin V and Propidium Iodide (Biolegend); Reverse transcription kit, DRR037A, and quantitative PCR kit, DRR820A (Takara); RIPA cell lysis buffer; BCA protein quantification kit (Bi-yun-tian, Shanghai); psiRNA-hTLR9 (Invivogen); RNA extraction kit (mirVana miRNA extraction kit, Ambion); *E. coli* DH5a competent cells (TransGen Biotech, Beijing).

### Methods

#### Podocyte culture and treatment

Podocytes were grown at 33 °C and switched to and incubated at 37 °C for 10–14 days. Cells were serum starved for 12 h before PAN treatment. Cells were treated with 25–100 μg/ml PAN for various time (3 h, 6 h, 12 h, and 24 h), followed by immunoblotting, qRT-PCR or flow cytometry analysis.

#### Immunoblotting

After the treatment, podocytes were washed with cold PBS and then lysed with 150 μl of RIPA buffer containing proteinase inhibitors cocktail (Roche) and phosphatase inhibitors. The lysates were incubated on ice and then centrifuged 12,000 g for 15 min at 4 °C. The supernatants were transferred to fresh tubes and then subjected to protein concentration measurement with BCA protein kit (Bio-Rad). After mixed with loading buffer, the samples were boiled at 98 °C for 5 min. 10% or 8% SDS-PAGE was used to fractionate the samples, and semi-dry transfer system (Bio-rad) was used to transfer the protein from the gel to PVDF membrane. The blot was blocked with 5% milk in TBST solution (20 mM Tris-HCl, PH 7.14, 150 mM NaCl, 0.1% Tween-20) for 60 min at room temperature, and then incubated with antibody overnight at 4 °C. After washed with TBST for 3 times, the blot was incubated with HRP-labeled secondary antibody for 1 h at room temperature. After washed, ECL system (Millipore) was used to detect the protein.

#### qRT-PCR analysis of gene expression

Total RNA of podocytes was prepared by using mirVana miRNA extraction kit (Ambion). Reverse transcription was performed using the kit from TakaRa (DRR037A). The primers of TLR9 were 5′-CCGTGACAATTACCTGGCCTTC-3′ (forward) and 5′-CAGGGCCTTCAGCTGGTTTC-3′ (reverse); IL12 primers: 5′-GGCCGTCAGCAACATGCTCCA-3′ (forward) and 5′-GGCACAGGGCCATCATAAAAGAGGT-3′ (reverse); 18 s rRNA: 5′TTCTCGATTCCGTGGGTGG-3′ (forward) and 5′-AGCATGCCAGAGTCTCGTTC-3′ (reverse). SYBR Green dye was used in the qPCR. The thermal condition was 95 °C/30 s for denature, followed by 40 cycyles of 95 °C/5 s − 60 °C /30 s on ABI 7900HT Fast Real time System. Threshold cycle (CT) values were determined and the relative abundance of the mRNA was calculated with the formula 2^−△△Ct^.

#### Apoptosis analysis by flow cytometry

After treatment, cells were stained with Annexin V and PI or 7-AAD following the manufactures′ instruction (Biolegend) and then subjected to flow cytometry analysis (FACS ARIA, BD).

#### siRNA silencing

TLR9 siRNA expressing plasmid, psiRNA-hTLR9, and control plasmid, psiRNA-h7SK, were transfected into podocytes using LipofectAMINE 2000. 24 h later, PAN was added to treat the cells for 24 h. The cells were then collected for the analyses of qPCR, immunoblotting,or flow cytometry following Annexin V staining.

#### TLR9 and DNase 2 overexpression in podocytes

To overexpression TLR9 or DNase 2 in cultured podocytes, TLR9 or DNase 2 expression plasmid, pUNO1-hTLR9 (Invivogen), or DNASE human cDNA (origene), was transfected into cultured podocytes using LipofecTAMINE 2000 (Invitrogen) following the method in the manual instruction.

#### Luciferase reporter assay

pNFκB-luc (beyotime) containing luciferase coding region downstream a NFκB-responsive promoter was transfected by LipofecTAMINE 2000 into podocytes. Twenty-four hours later, cell lysates were prepared and subjected to luciferase assays using the Dual-Luciferase Report Assay System (Promega). The firefly luciferase activities were normalized to the corresponding Renilla luciferase activities.

#### Animal study

We treated rats with PAN and performed urinary protein measurement, glomeruli isolation and RNA preparation, renal tissue immunohistochemical staining. All experiments followed the methods described^32^.

#### Human FSGS kidney samples

FSGS cases were diagnosed on the basis of renal biopsies performed at the National Clinical Research Center of Kidney Diseases of the Nanjing University School of Medicine. Kidney biopsies from patients with FSGS and normal kidney tissues from nephrectomy patients were obtained from this center′s renal biorepository. Human renal biopsies were fixed in formalin, embedded in paraffin and sectioned for IHC staining for TLR9 following the methods described^33^.

#### Study approval

The protocol of the use of biopsies from patients with FSGS and nephrectomized tissues was approved by the local committee on human subjects at Jinling Hospital, Nanjing University School of Medicine (2012GJJ-034), and the methods were carried out in accordance with the approved guidelines. Written informed consent was provided by all patients. All animal protocols and procedures were approved by the IACUC of Jinling Hospital, and the methods were carried out in accordance with the approved guidelines.

#### Statistical analysis

All experiments were performed for at least three times, and the results were given as mean ± SD. SPSS18.0 software was used for the statistical analyses. 2-tailed Student’s t test was used for comparison between two groups, and p < 0.05 was considered to be significantly different.

## Additional Information

**How to cite this article**: Bao, W. *et al*. Toll-like Receptor 9 Can be Activated by Endogenous Mitochondrial DNA to Induce Podocyte Apoptosis. *Sci. Rep.* **6**, 22579; doi: 10.1038/srep22579 (2016).

## Supplementary Material

Supplementary Information
