# Supplementary material for: Toll-like Receptor 9 Can be Activated by Endogenous Mitochondrial DNA to Induce Podocyte Apoptosis
Source: Sci Rep. 2016 Mar 3;6:22579. doi: 10.1038/srep22579 (PMC4776276; doi:10.1038/srep22579)
Supplement: Supplementary Information [file srep22579-s1.doc]

**Supplementary Information**

**Toll-like Receptor 9 Can Be Activated By Endogenous Mitochondrial DNA To Induce Podocyte Apoptosis**

Wenduona Bao^#^, Hong Xia^#^, Yaojun Liang^#^, Yuting Ye, Yuqiu Lu, Xiaodong Xu, Aiping Duan, Jing He, Zhaohong Chen, Yan Wu, Xia Wang, Chunxia Zheng, Zhihong Liu, Shaolin Shi^*^


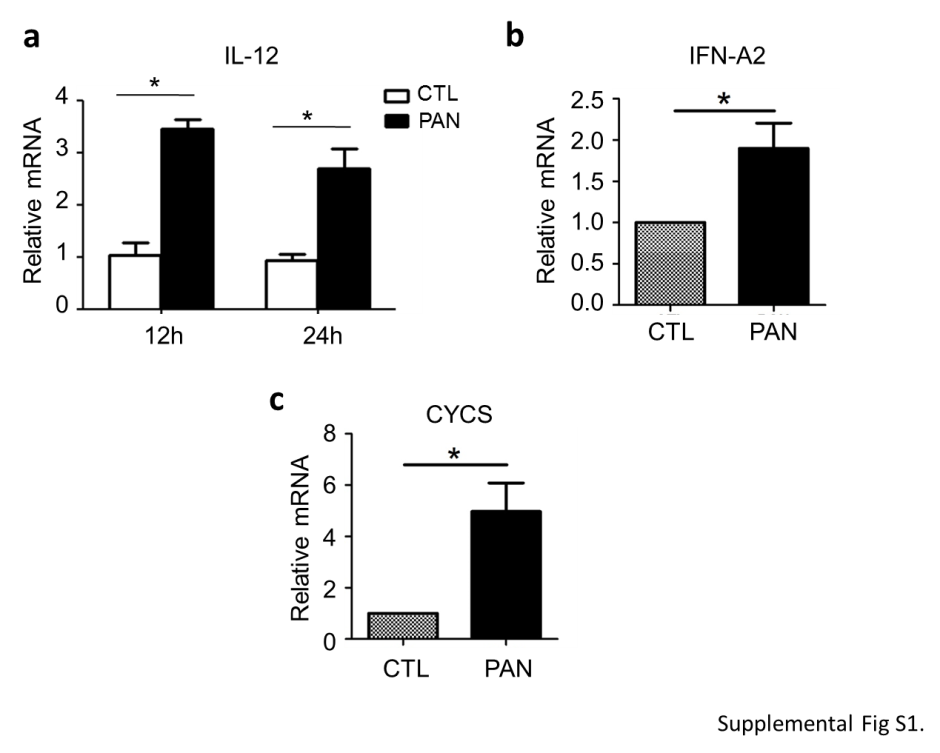


**Supplementary Figure S1**. qPCR analysis shows that PAN upregulated mRNA expression of IL-12 (**a**) , IFN-A2 (**b**), and CYCS (**c** ). Data are expressed as the mean± SD of three independent experiments. *P<0.05.


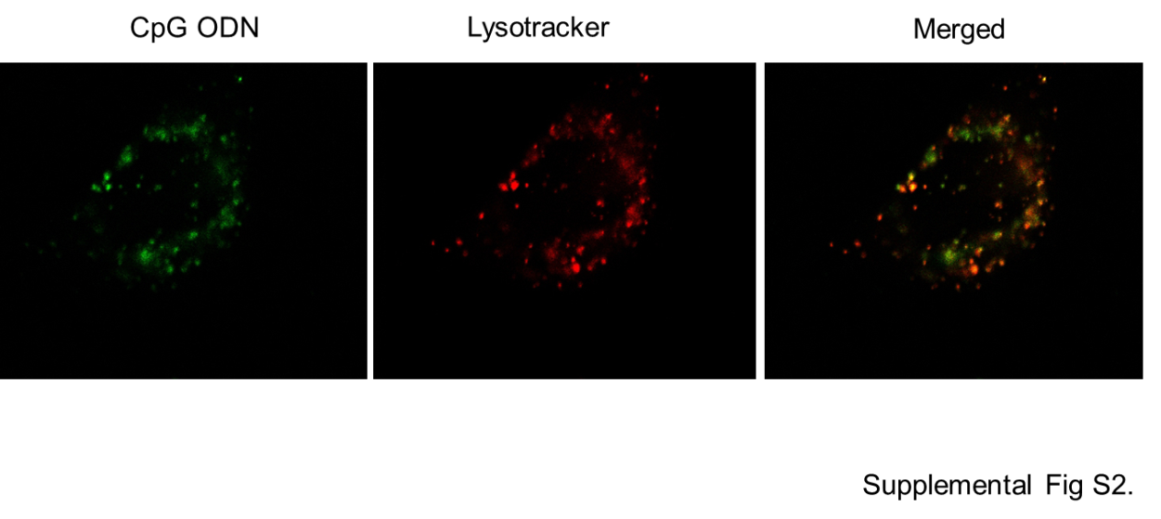


**Supplementary Figure S2.** Colocalization of uptaken CpG ODN and endolysosome marker, lysotracker in cultured podocytes.


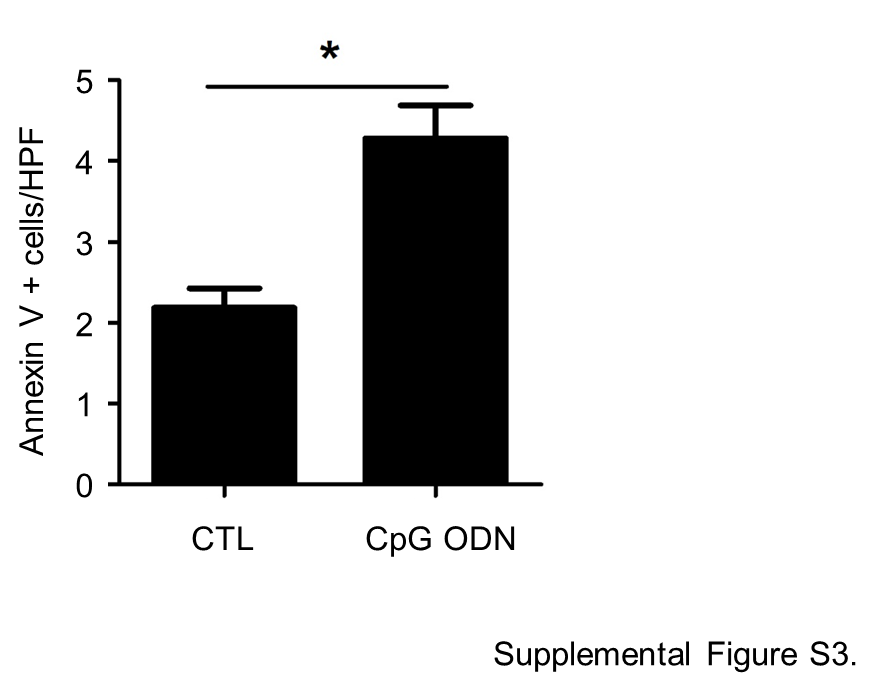


**Supplementary Figure S3.** CpG ODN treatment increased apoptosis in cultured podocytes. Podocytes were treated with 10 µg/ml CpG ODN for 24h, and stained with Annexin V following the method described [1]. The Annexin V positive cells were counted under a fluorescence microscope. A total of 50 high power fields (200X) were examined in each case, and a mean was obtained. Data are expressed as the mean± SD of three independent experiments. *P<0.05 versus untreated control.


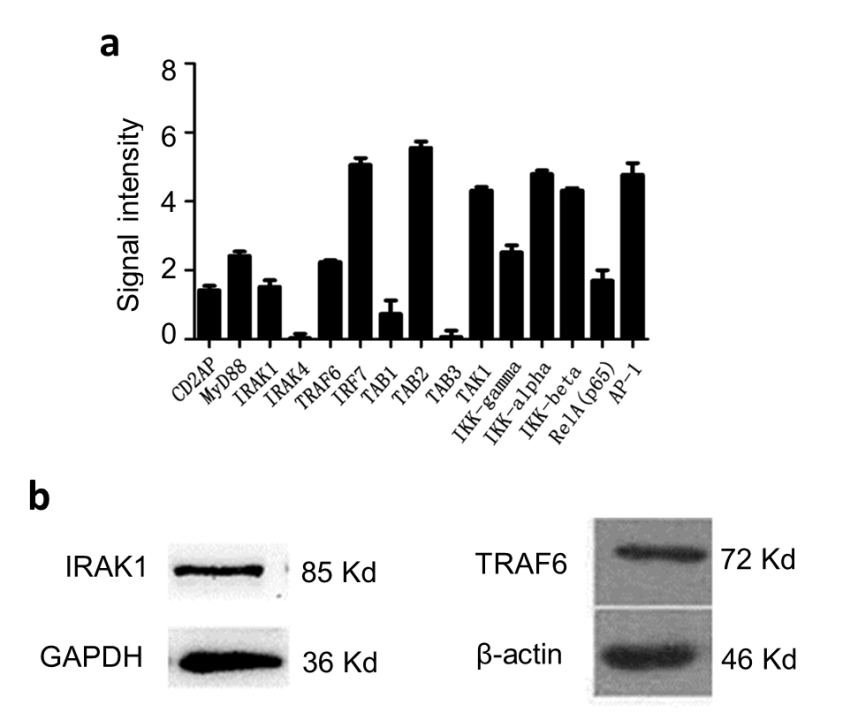


**Supplementary Figure S4**. Analysis of TLR9 signaling components in the immortalized human podocytes. **a**. microarray gene expression profile detected significant expression of TLR9 signaling components in the cells. **b**. Immunoblotting confirmed the presence of the protein IRAK1 and TRAF6.


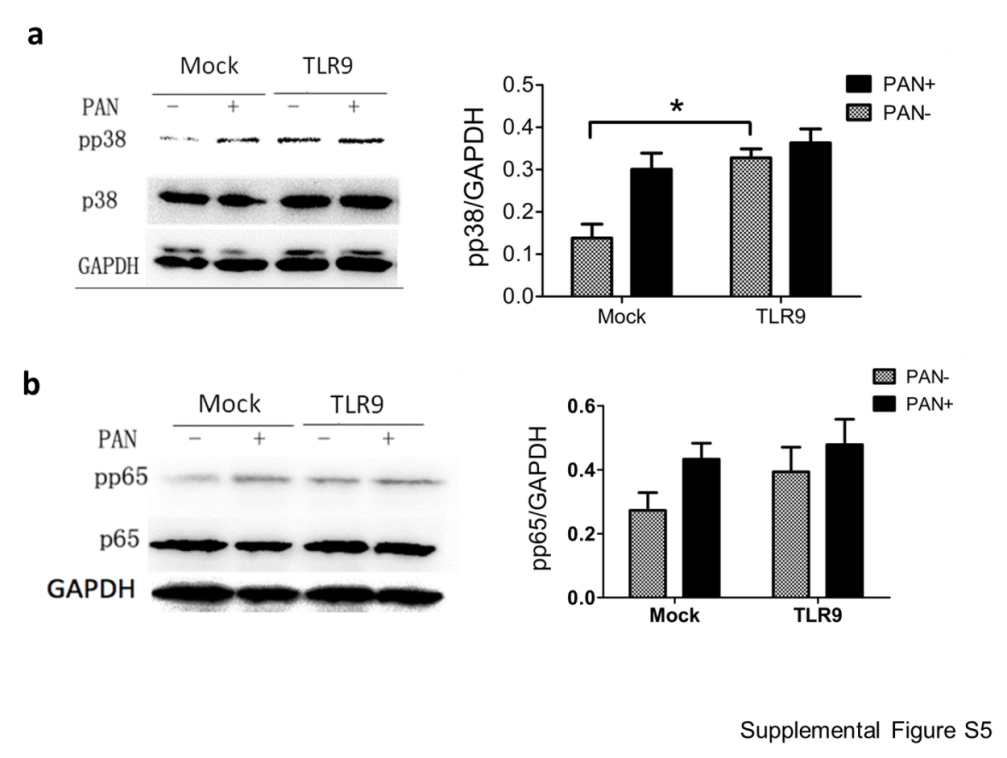


**Supplementary Figure S5**. The effect of TLR9 overexpression on p38 and p65 phosphorylation. **a**. Immunoblotting showing a significant increase of pp38 by TLR9 overexpression in the absence of PAN but no further increase of pp38 in the presence of PAN. *P<0.05. **b**. pp65 was increased in the podocytes overexpressing TLR9 in the absence of PAN. The bar graphs on the right in Panel b and c show the quantification of the blots from three independent experiments. *P <0.05.


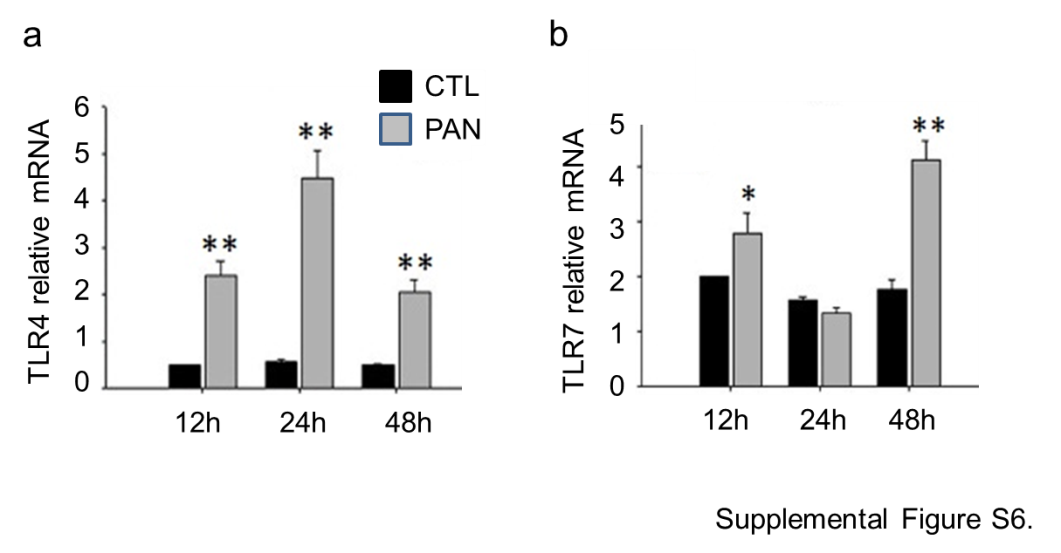


**Supplementary Figure S6.** qRT-PCR analysis showing that TLR4 (**a**) and TLR7 (**b**) were upregulated by PAN in cultured podocytes. All data are expressed as the mean± SD of three independent experiments. *P<0.05 and **P<0.01 versus untreated controls.


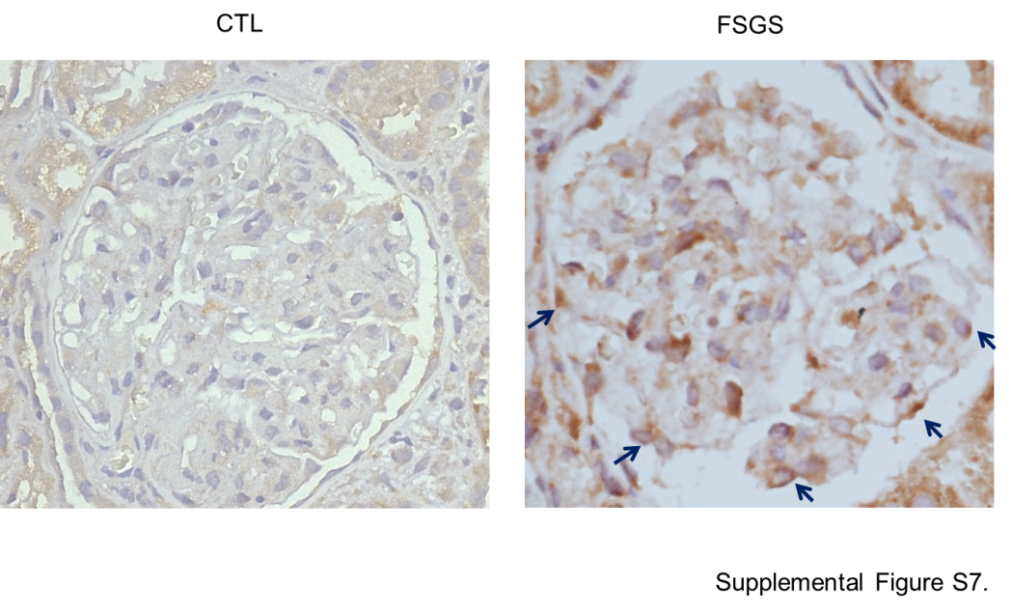


**Supplementary Figure S7.** Immunohistochemical staining of TLR9 in renal biopsies of a FSGS patient and normal control. It shows that TLR9 was not expressed in podocytes of normal control but significantly expressed in podocytes of a FSGS patient (black arrows).

**References:**

1. Niranjan, T., et al., *The Notch pathway in podocytes plays a role in the development of glomerular disease.* Nat Med. 2008, 14(3): p.290-8.
